# Assessment of levels of asthma control among adult patients with asthma at Chitungwiza Central Hospital, Zimbabwe

**DOI:** 10.1186/s13223-020-0405-7

**Published:** 2020-02-04

**Authors:** Pisirai Ndarukwa, Moses John Chimbari, Elopy Sibanda

**Affiliations:** 1 College of Health Sciences, School of Nursing and Public Health, University of KwaZulu Natal, 1st Floor, George Campbell Building, Howard College Campus, UKZN, Durban, 4000 South Africa; 2 Asthma, Allergy and Immune Dysfunction Clinic, 113 Kwame Nkrumah Ave, Harare, Zimbabwe

**Keywords:** Asthma, Control of asthma, Asthma Control Questionnaire (ACQ), Spirometry, Zimbabwe

## Abstract

**Background:**

The management of the reversible airways obstruction forms the cornerstone of quality asthma management. The aim of this study was to assess the level of control for asthma in adult patients, using a cross sectional study design. The assessment of control for asthma was based on the ACQ.

**Methods:**

We conducted a cross-sectional study to measure the level of control for asthma among patients with asthma who volunteered and were reporting to Chitungwiza Central Hospital. We interviewed and conducted spirometry (lung function testing) on 400 adult patients with asthma. We used the ACQ questions to interview patients. A trained health care provider performed spirometry using the Koko Legend spirometer after meeting all the ambient conditions as outlined in the American Thoracic Society guidelines.

**Results:**

Our study assessed levels of asthma control among 400 adult patients with physician-diagnosed asthma. The results showed that 248 (62%) participants had uncontrolled asthma. The median age of the adult patients who had uncontrolled asthma was 35 years (IQR: 27–44). Using the clinical practice cut-point of 0.75 for controlled asthma, only 152 (38%) were controlled, while 72 (18.8%), 50 (12.5%) and 123 (30.7%) were mildly uncontrolled, moderately uncontrolled and very uncontrolled respectively. Among participants who were widowed had uncontrolled asthma (p = 0.003) while most of the married 103 (67.8%) had controlled asthma (p = 0.018). The findings of the study showed that all the items on the ACQ were significantly different in asthma mean scores (p ≤ 0.0001).

**Conclusion:**

We concluded that most asthma patients that participated in the study were uncontrolled. We therefore, recommend an evaluation of factors associated with poor asthma control in order to improve asthma care and achieve good asthma control outcomes.

## Background

Asthma is defined as a heterogeneous disease, usually characterized by chronic airway inflammation and reversible airway obstruction [1]. It affects an estimated 300 million people of all ages worldwide [1]. It is thus important to manage this reversible airways obstruction as it forms the cornerstone to quality asthma management [1, 2]. Asthma control is the extent to which numerous clinical presentations of asthma are reduced or removed by asthma treatment [3]. The control of asthma ensures that patients with asthma live their daily lives as healthy people do [3].

In order to achieve good control of asthma, the treatment should result in day and night symptoms less than 4 times per week, improve performance of daily activities, reduce asthma attacks, and that reliever treatment be less than four times per week [4]. In addition, good quality management of asthma requires that the patient adheres to the prescribed treatment. The treatment should include the use of controllers AND quick RELIEVERS [5, 6]. Glucocorticosteroids, β2-agonists and anti-leukotriene are the medical treatments most commonly recommended for the asthma patients. The patients need to have a good knowledge of the asthma disease in order to be able to follow the treatment regimens and to use different inhalers; and to avoid factors that can cause or trigger complications. Often lifestyle changes such as the need for daily medication, stopping habits of smoking, identifying and avoiding triggers as well as regular physical exercise, are necessary to achieve asthma control [7].

According to Nieuwenhof et al. [8], a high number of adult asthma patients were not adequately controlled despite effective asthma treatments being available. Further, studies have indicated that the major reason for poor asthma control is patients’ failure to adhere to their treatment [9]. Assessing asthma control level could give an insight into the level to which quality of care is rendered to the asthma patients. One assessment tool that has been widely used to ascertain the levels of asthma control is the Asthma Control Questionnaire (ACQ)© which was developed to assess the effectiveness in quality management of asthma [10]. The ACQ has undergone a rigorous validation process [10] and has been standardized and validated in a number of settings [11] which include South Africa and European countries studies [12–14] have shown that the ACQ questionnaire is valid for measuring asthma control and provide a strong assessment tool that can be used in clinical practice and research. However, although there have been studies assessing levels of asthma control [3, 14, 15] elsewhere, there have been no documented studies on levels of asthma control within the Zimbabwean population. The aim of this study therefore, was to assess level of asthma control in a Zimbabwean adult asthma population attending Chitungwiza Central Hospital.

## Methods

### Study design and setting

We conducted a descriptive cross sectional survey using an ACQ to assess the levels of asthma control among adult patients attending a research clinic at Chitungwiza Central Hospital in Zimbabwe. The study used those (physician-diagnosed asthma patients) participants who volunteered. Chitungwiza Central Hospital is situated in Chitungwiza town which is approximately 30 km south east of Harare. The hospital has a bed capacity of 500 beds including general, specialised, maternity and emergency care beds. There is a causality department where patients presenting with acute asthma attack are managed. Complicated cases of asthma including *status asthmaticus* are managed in the high dependency unit.

### Sample size determination and sampling techniques

A sample size of 384 was calculated using the Dobson formula: $n = \frac{{z^{2} pq}}{{e^{2} }}$ [16], which was then adjusted to 400 to cater for large volumes of patients who were reporting to the study clinic.

Where “n” is the sample size, “z” is the standard normal variable, “p” is the expected proportion in the population (50% was assumed since the prevalence of asthma was unknown) and “e” is the absolute error or precision. An error margin of 5% at 95% confidence interval was sought in this study. We conveniently sampled 400 physician-diagnosed asthma patients at Chitungwiza Central Hospital. All patients with physician-diagnosed asthma were evaluated in this study and these reported at an established research study clinic. These patient with physician-diagnosed asthma were all of the African ethnic group. This research study clinic was established solely for this study. Recruitment of large number of patients in a short period was achieved through intense community sensitization of the project prior to the recruitment period. Asthma flyers were distributed to local health care facilities and community-meeting points to ensure that participants are sensitised of the study (see Additional file 1). We used the community engagement approach to mobilise participants into this study. The community engagement aims to increase the participation of community stakeholders in research [17]

### Data collection using the ACQ©

We adopted the Asthma Control Questionnaire (ACQ South African English Version) to measure asthma control levels [10]. In addition, we used a spirometer to measure the Forced Expiratory Vital at 1 s (FEV1) as objective measure of asthma control. Data were collected from 29 November 2018 to 16th of December 2018. The health professionals (nurses) interviewed all the 400 physician-diagnosed asthma patients during the data collection period. The patients were asked to recall their symptoms during the previous week and to respond to the first 6 questions (night-time waking, symptoms on waking, activity limitation, shortness of breath, wheeze, and use of rescue treatment) on a 7-point scale from 0 (no impairment) to 6 (maximum impairment). The trained instructor performed the spirometry on all patients in order to respond to the 7th item question which required similar scale as the other ACQ questions.

### Measures

We collected data using the ACQ© [11] and the spirometer. The ACQ is a 7-item questionnaire, which includes five items on the most important asthma symptoms, one item on the use of the rescue bronchodilator and the last item on the FEV1% predicted. Thus the health professionals (nurses) asked six questions from the ACQ to the participants with the last item requiring that a spirometry be performed. ACQ score is the mean of the 7 items, with scores between 0 (totally controlled) and 6 (severely uncontrolled).

The 6-self report items on the ACQ (see Additional file 2: Appendix S1).

Spirometry was ascertained through the use of a Koko legend spirometer which is a diagnostic breathing test that measures the volume and flow of air that can be inhaled and exhaled by the lungs and is reliable to differentiate between obstructive and restrictive lung diseases [18]. A qualified instructor using exemplary spirometry demonstrations did all the spirometry tests.

### Procedures

Items from the ACQ and demographic data were fed into the KoBoCollect Toolbox before actual data collection commenced. The KoBoCollect is an open source platform that is utilised when collecting and analysing data [19]. Trained nurses administered the ACQ after obtaining written consent from all participants. All interviews were held in a private room.

Spirometry was performed to establish the FEV1%. The spirometer was calibrated daily and after change in ambient conditions in accordance with the American Thoracic Society (ATS) guidelines [20]. All the tests were performed from a standing height and patients took an average 8–11 min to produce a minimum of 3 flow-volume and volume-time curves. The curves were checked for quality of the peak and expiration phase by inspecting:

1. The presence of an instantaneous start and a rapid rise in expiratory flow. Thus, a flat peak indicates lack of effort at the beginning of the spirometry manoeuvre.
2. Presence of a smooth continuous downward expiration with no evidence of erratic exhalation, cough or glottis closure.
3. An expiration time of ≥ 6 s in adults 18 years and above.
4. A plateau on the volume-time curve without an abrupt end in the expiration phase [20–22].

The best reading of the three acceptable and repeatable efforts calculated as the largest sum of FVC and FEV_1_ according to the time of expiration was then recorded [23].

### Data analysis

We used Stata Version 13 package for analyzing data. Descriptive data analysis was performed for demographic data reporting differences in proportions (p-value < 0.05) by asthma control status. Further we used the clinical practice cutoff point to differentiate between asthma control levels [24]. Analysis of variance (ANOVA) was then used to compare mean scores for different asthma control levels by item questions on the ACQ. The Bonferroni analysis cut off point of 0.75 with a Minimal Important Difference of 0.5 to detect any minimal change in asthma control was used to determine the different asthma controls level.

### Interpretation of ACQ Data

Using the ACQ, we defined the Minimal Important Difference (MID) into four groups [Group 1 = Controlled asthma (ACQ ≤ 0.75), Group 2 = mildly uncontrolled asthma (0.75 > ACQ ≤ 1.25), Group 3 = moderately uncontrolled asthma (1.25 > ACQ ≤ 1.75) and Group 4 = very uncontrolled asthma (ACQ ≥ 1.57)] as described by Juniper et al. [24]. The cut off points of ACQ < 0.75 for controlled asthma in the clinical practice was used to distinguish the well-controlled from uncontrolled asthma [24]. The MID for the ACQ of 0.5 on the 7-point scale was used as determined in other studies [25, 26].

### Ethical approval and consent to participate

We obtained ethics approval to conduct the study from Medical Research Council of Zimbabwe (A/2352) and Biomedical Research Ethics Committee of the University of KwaZulu-Natal (BE613/18). Gatekeepers’ permission was sought from Chitungwiza Hospital as well as Ministry of Health and Child Care, Zimbabwe. We also got permission from Prof Juniper to use the copyrighted Asthma Control Questionnaire (ACQ) (see attached Additional file 2: Appendix S2) with specific instructions that we could not retype or translate it into local language. Nursing professionals who had knowledge of how to ask these questions to the patients without having to translate the ACQ and retaining the meaning of the questions administered the questionnaire.

## Results

A total of 400 participants 261 (65.3% females) with asthma participated in the study. The majority 245 (61.2%) were married, 97 (24.3%) were single while 36 (9%) and 22 (5.5%) were widowed and divorced respectively. There were significant differences between males and females with controlled asthma. The results of the study further showed a significant difference between males and females with uncontrolled asthma (p ≤ 0.001). Those participants who attained secondary level of education were 159 (39.7%). Those who had a primary, college and university level of education were 60 (15%), 127 (31.8%) and 54 (13.5%) respectively. There are significant differences between participants who attended primary school and those that had secondary and college school education regardless of asthma control status (p = 0.001). With regards religion, 163 (40.7%) reported to be Pentecostal, 100 (25%) were Apostolic, and 79 (19.8%) were Protestants. Slightly above half 201 (50.3%) reported to be employed. The majority 272 (68%) resided in urban areas, and the least 26 (6.5%) were from surrounding farms.

Among those 152 (38%) who had controlled asthma, the Pentecostals 68 (44.7%) were the most, followed by the Apostolic. However, for uncontrolled asthma, 95 (38.3%) and 68 (27.4%) were the Pentecostal and protestants respectively. Among the Apostolic, more had significantly controlled asthma (p = 0.001). However, the protestants had a significantly uncontrolled asthma (p < 0.001).

On analysis, there were more study participants 248 (62%) who had uncontrolled asthma. The median age for the participants was 33 years (IQR: 25.5–41 years) in those with controlled asthma and was 35 years (IQR: 27–44 years) in those with uncontrolled asthma. Of the 152 participants who were classified to have controlled asthma, more than half 103 (67.8%) were married. Of the 248 participants classified to have uncontrolled asthma, 30 (12.1%) were widowed.

From a total of 152 participants who had controlled asthma, 51 (33.6%) apostolic sect members reported controlled asthma. On the contrary, from the 248 participants who reported to be protestants, 68 (27.4%) had uncontrolled asthma. The distribution of apostolic participants was different to Pentecostal and protestants participants for all groups (p < 0.001). A Chi-square test was performed to identify an association (p-value < 0.05) between participants’ demographic characteristics and asthma control status. The distribution of participants by area of residence was consistent regardless of asthma control status (p < 0.001). Participants from urban areas were different from peri-urban, rural and those from farms in both control groups (controlled and uncontrolled). The distribution showed significant differences between employed and unemployed participants in both controlled asthma and uncontrolled asthma participants (see Table 1).

**Table 1:** Participants’ demographic characteristics by asthma control status using the clinical practice cut off point of 0.75 for controlled asthma

Characteristic	Totals	Controlled asthma (n = 152) ACQ score (< 0.75)		Uncontrolled asthma (n = 248) ACQ score (> 0.75)		p-value^1^	p-value^2^
			p-value		p-value		
Median age (IQR)		33 (25.5–41)		35 (27–44)			
Sex							
Male	139 (34.8%)	51 (33.6%)	1	88 (35.5%)	1	0.349	0.694
Female	261 (65.2%)	101 (66.4%)	< 0.001	160 (64.5%)	< 0.001	0.651	
Level of education							
Primary	60 (15.0%)	19 (12.5%)	1	41 (16.5%)	1	0.137	0.724
Secondary	159 (39.7%)	63 (41.4%)	< 0.001	96 (38.7%)	< 0.001	0.293	
College	127 (31.8%)	50 (32.9%)	< 0.001	77 (31.0%)	< 0.001	0.351	
University	54 (13.5%)	20 (13.2%)	0.428	34 (13.8%)	0.201	0.438	
Marital status							
Married	245 (61.2%)	103 (67.8%)	1	142 (57.2%)	1	0.018**	0.012*
Single	97 (24.3%)	38 (25.0%)	< 0.001	59 (23.8%)	< 0.001	0.392	
Widowed	36 (9.0%)	6 (4.0%)	< 0.001	30 (12.1%)	< 0.001	0.003**	
Divorced	22 (5.5%)	5 (3.2%)	< 0.001	17 (6.9%)	< 0.001	0.065	
Religion							
Apostolic	100 (25.0%)	51 (33.6%)	1	49 (19.8%)	1	0.001**	< 0.001*
Pentecostal	163 (40.7%)	68 (44.7%)	0.024	95 (38.3%)	< 0.001	0.102	
Protestants	79 (19.8%)	11 (7.2%)	< 0.001	68 (27.4%)	0.023	< 0.001**	
Others (traditional Africans)	58 (14.5%)	22 (14.6%)	< 0.001	36 (14.5%)	0.059	0.506	
Employment status							
Employed	201 (50.3%)	87 (57.2%)	1	114 (46.0%)	1	0.014**	*0.029*
Unemployed	199 (49.7%)	65 (42.8%)	0.006	134 (54.0%)	0.037	0.014	
Area of residence							
Urban	272 (68.0%)	118 (77.6%)	1	154 (62.1%)	1	< 0.001**	*0.004*
Peri-urban	44 (11.0%)	7 (4.6%)	< 0.001	37 (14.9%)	< 0.001	< 0.001**	
Rural	58 (14.5%)	21 (13.8%)	< 0.001	37 (14.9%)	< 0.001	0.381	
Farms	26 (6.5%)	6 (4.0%)	< 0.001	20 (8.1%)	< 0.001	0.050**	

Italic values indicate significance of p-value (p < 0.05)

^**^Statistically significant; p-value^1^: student t-test for asthma control proportions between participants with controlled and uncontrolled asthma; p-value^2^: chi-square group test for the association between asthma control status and demographic characteristics

Table 2 compares the ACQ asthma mean scores for the 7 item questions. Using the ACQ asthma control mean scores, 152 (38%) participants had a controlled asthma (FEV1%: ≥ 80). Those with a mild uncontrolled asthma (FEV1%: 60–79) contributed to 72 (18.8%), while 50 (12.5%) were moderately uncontrolled (FEV1%: 51–59). The remaining 123 (30.8%) had very uncontrolled asthma (FEV1%: ≤ 50). All the 7 ACQ items indicated significant differences in asthma mean scores (p < 0.001). Bonferroni analysis to distinguish between paired groups using ANOVA showed that all the identified questions (1–6) were significantly different. The predicted FEV1% for the mean scores for the controlled asthma group against moderately uncontrolled and very uncontrolled asthma groups were significantly different (p ≤ 0.001). The predicted FEV1% for the mean scores for the mildly uncontrolled asthma group against moderately uncontrolled and very uncontrolled asthma groups were significantly different (p = 0.003).

**Table 2:** Comparison of the ACQ asthma control mean scores against the Minimal Important difference of 0.5

Item	Well-controlled (n = 152) (ACQ ≤ 0.75) *Mean ACQ score (SD)*	Mildly uncontrolled (n = 75) (0.75 > ACQ ≤ 1.25 *Mean ACQ score (SD)*	Moderately uncontrolled (n = 50) (1.25 > ACQ ≤ 1.75) *Mean ACQ score (SD)*	Very uncontrolled (n = 123) (ACQ > 1.75) *Mean ACQ score (SD)*	p-value
Q1	0 (0)	1.067 (0.3002)	1.46 (0.503)	2.67 (1.206)	< 0.001
Q2	0.03 (0.195)	1.07 (0.379)	1.46 (0.503)	2.85 (1.15)	< 0.001
Q3	0.01 (0.114)	1.01 (0.115)	1.46 (0.613)	2.81 (1.20)	< 0.001
Q4	0.026 (0.198)	1.03 (0.328)	1.46 (0.542)	2.91 (1.109)	< 0.001
Q5	0.026 (0.198)	0.96 (0.257)	1.5 (0.505)	2.91 (1.201)	< 0.001
Q6	0.026 (0.198)	0.96 (0.257)	1.5 (0.505)	2.83 (1.084)	< 0.001
Q7	0.54 (1.02)	0.44 (0.81)	1.76 (1.86)	3.85 (1.95)	< 0.001

p-values for mean comparisons in the four asthma severity groups

## Discussion

This study aimed to measure the level of asthma control in a Zimbabwean adult asthma population attending the research study clinic at a central hospital. Results indicated that most patients had uncontrolled asthma. Our results resonate those reported in South Africa where 47.2% of patients were controlled [27]. In addition, studies from high-income countries have also highlighted this trend with a multi-country study conducted in five European countries indicating that more than half (56.6%) of treated asthmatics were not well controlled which is in tandem with our findings [28].

Such results are important for a number of reasons. For instance, the high levels of uncontrolled asthma present opportunities for healthcare providers to institute interventions, which will improve asthma treatment. Second, there will be need for more studies to explore patient related and health service related factors contributing to uncontrolled asthma in resource-limited settings such as ours.

Our study has shown that a better control of asthma was amongst the Pentecostal sect members. This could be as a result of the fact that members of the Pentecostal religion engage in medical pluralism which allows them to take medication and faith healing. Further, our study has shown that members of apostolic sect members had a better control of asthma compared to Protestants despite the known fact that apostolics do not normally seek care at health facilities. This is especially interesting to note that a better control of asthma was amongst the apostolic sect participants compared to the Protestants. Within our setting, the apostolic do not provide shelter for their congregants and spend most of their time under trees and in open spaces, which are dusty, and wind-blown whilst the Protestants provide shelter for their congregants and emphasize on clean environments. This finding is further supported by the hygiene hypothesis [15]. Further the result in this study is surprising given that the Protestants whose level of utilization of hospital services is better than that of the apostolic sects in Zimbabwe. Therefore there could be need to study what the apostolic are doing which lead to better asthma control.

Our results showed that those employed had controlled asthma while those that were unemployed had uncontrolled asthma. Such results may be explained in part, by the fact that patients who are likely to be employed may be better informed and have a better quality of life which may lead them to be more compliant to treatment regiments and recommendations from healthcare personnel. Additionally employed patients may be in a better position to afford healthcare services in a setting such as Zimbabwe where health care costs are expensive for those without a steady income [29]. Previous studies have shown that employment status is associated with controlled/uncontrolled asthma [30, 31].

Results from the current study indicated that being married was associated with having well-controlled asthma which might be due to the fact that married couples could be having more social support. However, our results further elaborated that those that were widowed, separated or divorced were likely to be associated with uncontrolled asthma. This may be due to the fact that widowhood and to some extent being divorced, erodes social and financial support. Similar to our findings, researchers from India reported that being widowed was associated with more adverse health outcomes for patients with chronic conditions [32]. Furthermore, our results concur with those of Zhang et al. [33] that showed that married individuals were likely to have better survival outcomes because their spouses cared for them and encouraged them to have a positive outlook. There is thus need to strengthen health promotion interventions targeting groups such as the widowed in an effort to improve the quality of life of asthma patients.

Another key determinant of poor asthma control that emerged from the results of this study pertain to the participants’ area of residence. For instance residing in urban areas appeared to be protective against uncontrolled asthma with those participants from farming communities having more uncontrolled asthma levels. Reasons for such discrepancies are however not clear, and the authors hypothesize that it could be due to differences in socio-economic statuses with urban dwellers being more affluent and hence could be having more contact with healthcare providers in addition to proximity of the hospitals and other health centres within their neighbourhoods. Our study findings concurred with the findings of a study by Braido, et al. [30] and Ghanname et al. [34] who showed that those staying in urban setting were more likely to be asthma controlled and those staying in the rural/villages being asthma uncontrolled. However, care must be taken in interpreting this result as our study was urban based and as such rural patients could have been misrepresented as they were fewer than their urban counterparts. Future studies could potentially compare patients attending rural health centres and urban ones.

In this study asthma control is significantly different within asthma control groups. The results may reflect the need to step up asthma treatment according to level of asthma control if we are to achieve best asthma control.

### Limitations

Although to the authors’ knowledge this is one of the first studies to document asthma control levels in Zimbabwe, results of the study should be interpreted with caution as there were some limitations. We have taken note of the recruitment approach that was followed in this study as one of the limitations of this study. We distributed some flyers to advertise for participants who would come to participate in our study. This could have acted as a limitation in that more uncontrolled asthma patient accepted the call for participation in the study. The study was conducted in a predominantly urban area that makes extrapolation of the results to rural areas difficult. In this study, we did not distinguish between the different categories of asthma severity.

## Conclusion

Although good asthma control can be achieved for most patients, the reality in clinical practice based on this study, it seems to suggest that asthma remains poorly controlled. Our study concluded that the majority of asthma patients who were being treated at Chitungwiza Central Hospital in Zimbabwe had uncontrolled asthma. This indicates the need to improve quality of care to these patients if control of asthma is to be achieved. There is also need to carry out a study that does not follow a community engagement approach, as this might have lead to most of those with uncontrolled asthma reporting to the research study clinic. The use of ACQ may provide the health care providers with a good and efficient starting point to actively and timely detect patients with uncontrolled asthma symptoms in clinical practice.

## Supplementary information

**Additional file 1.** Asthma advertising flyer.

**Additional file 2**:Asthma Control Questionnaire and Copyright letter of permission. **Appendix S1.** Asthma Control Questionnaire. **Appendix S2.** ACQ letter of copyright permission.

## Data Availability

The datasets used and/or analyzed during the current study are available from the corresponding author on reasonable request.

## Abbreviations

ANOVA: Analysis of variance
ACQ: Asthma Control Questionnaire
ATS: American Thoracic Society
FEV1: Forced Expiratory Vital at 1 s
IQR: Interquartile range
MID: Minimal Important Difference
UKZN: University of KwaZulu-Natal
