# Supplementary material for: Assessment of levels of asthma control among adult patients with asthma at Chitungwiza Central Hospital, Zimbabwe
Source: Allergy Asthma Clin Immunol. 2020 Feb 4;16:10. doi: 10.1186/s13223-020-0405-7 (PMC7001265; doi:10.1186/s13223-020-0405-7)
Supplement: Supplementary file 1 — Additional file 1. Asthma advertising flyer. [file 13223_2020_405_MOESM1_ESM.docx]

**Additional file 1: Asthma advertising flyer.**

**Assessing level of control for asthma in adult patients with asthma at Chitungwiza Central Hospital.**


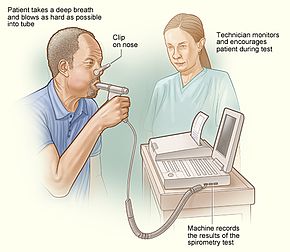
*****Spirometry machine*

This flyer is informing you of a study that you can volunteer to participate. The study seeks to assess level of asthma control at Chitungwiza Central Hospital, Zimbabwe. We will use the Asthma control questionnaire to assess level of asthma control. Further a spirometry will be performed free of charge during the study period. The Spirometry test will assist us to assess your level of asthma control.

Date: ……………………………………………………………..

Time: 7am-16:00pm

RSVP: Pisirai Ndarukwa

+263773012397

[papandarukwa@gmail.com](mailto:papandarukwa@gmail.com)

| **Asthma advertising flyer** |
| --- |
