# Supplementary material for: Assessment of levels of asthma control among adult patients with asthma at Chitungwiza Central Hospital, Zimbabwe
Source: Allergy Asthma Clin Immunol. 2020 Feb 4;16:10. doi: 10.1186/s13223-020-0405-7 (PMC7001265; doi:10.1186/s13223-020-0405-7)
Supplement: Supplementary file 2 — Additional file 2:Asthma Control Questionnaire and Copyright letter of permission. Appendix S1. Asthma Control Questionnaire. Appendix S2. ACQ letter of copyright permission. [file 13223_2020_405_MOESM2_ESM.docx]

Additional file 2 : Asthma Control Questionnaire and Copyright letter of permission.

Appendix S1 : Asthma Control Questionnaire.

E.F. JUNIPER ET AL.

ASTHMA CONTROL QUESTIONNAIRE©

Please answer questions 1±6.

Circle the number of the response that best describes how you have been during the past week

| 1. | On average, during the past week, how often were you **woken by your asthma** during the night? | 0  1 | Never  Hardly ever |
| --- | --- | --- | --- |
|  |  | 2 | A few minutes |
|  |  | 3 | Several times |
|  |  | 4 | Many times |
|  |  | 5 | A great many times |
|  |  | 6 | Unable to sleep because of asthma |
| 2. | On average, during the past week, how **bad were your asthma symptoms when you woke** up in the morning? | 0  1 | No symptoms  Very mild symptoms |
|  |  | 2 | Mild symptoms |
|  |  | 3 | Moderate symptoms |
|  |  | 4 | Quite severe symptoms |
|  |  | 5 | Severe symptoms |
|  |  | 6 | Very severe symptoms |
| 3. | In general, during the past week, how **limited were you in your activities** because of your asthma? | 0  1 | Not limited at all  Very slightly limited |
|  |  | 2 | Slightly limited |
|  |  | 3 | Moderately limited |
|  |  | 4 | Very limited |
|  |  | 5 | Extremely limited |
|  |  | 6 | Totally limited |
| 4. | In general, during the past week, how much **shortness of breath** did you experience because of you asthma? | 0  1 | None  A very little |
|  |  | 2 | A little |
|  |  | 3 | A moderate amount |
|  |  | 4 | Quite a lot |
|  |  | 5 | A great deal |
|  |  | 6 | A very great deal |
| 5. | In general, during the past week, how much of the time did you **wheeze**? | 0 | Not at all |
|  |  | 1 | Hardly any of the time |
|  |  | 2 | A little of the time |
|  |  | 3 | A moderate amount of the time |
|  |  | 4 | A lot of the time |
|  |  | 5 | Most of the time |
|  |  | 6 | All the time |
| 6. | On average, during the past week, how many **puffs of short-acting bronchodilator** (eg. Ventolin) have you used each day? | 0  1 | None  1±2 puffs most days |
|  |  | 2 | 3±4 puffs most days |
|  |  | 3 | 5±8 puffs most days |
|  |  | 4 | 9±12 puffs most days |
|  |  | 5 | 13±16 puffs most days |
|  | To be completed by a member of the clinic staff | 6 | More than 16 puffs most days |
| 7. | FEV1 pre-bronchodilator: ................................ | 0 | >95% predicted |
|  |  | 1 | 95±90% |
|  | FEV1 predicted ................................................ | 2 | 89±80% |
|  |  | 3 | 79±70% |
|  | FEV1 % predicted ...........................................  (Record actual values on the dotted lines and score the FEV1 % predicted in the next column) | 4  5  6 | 69±60%  59±50%  <50% predicted |

©The Asthma Control Questionnaire is copyrighted. It may not be changed, translated or sold (paper or software) without the permission of Elizabeth Juniper.


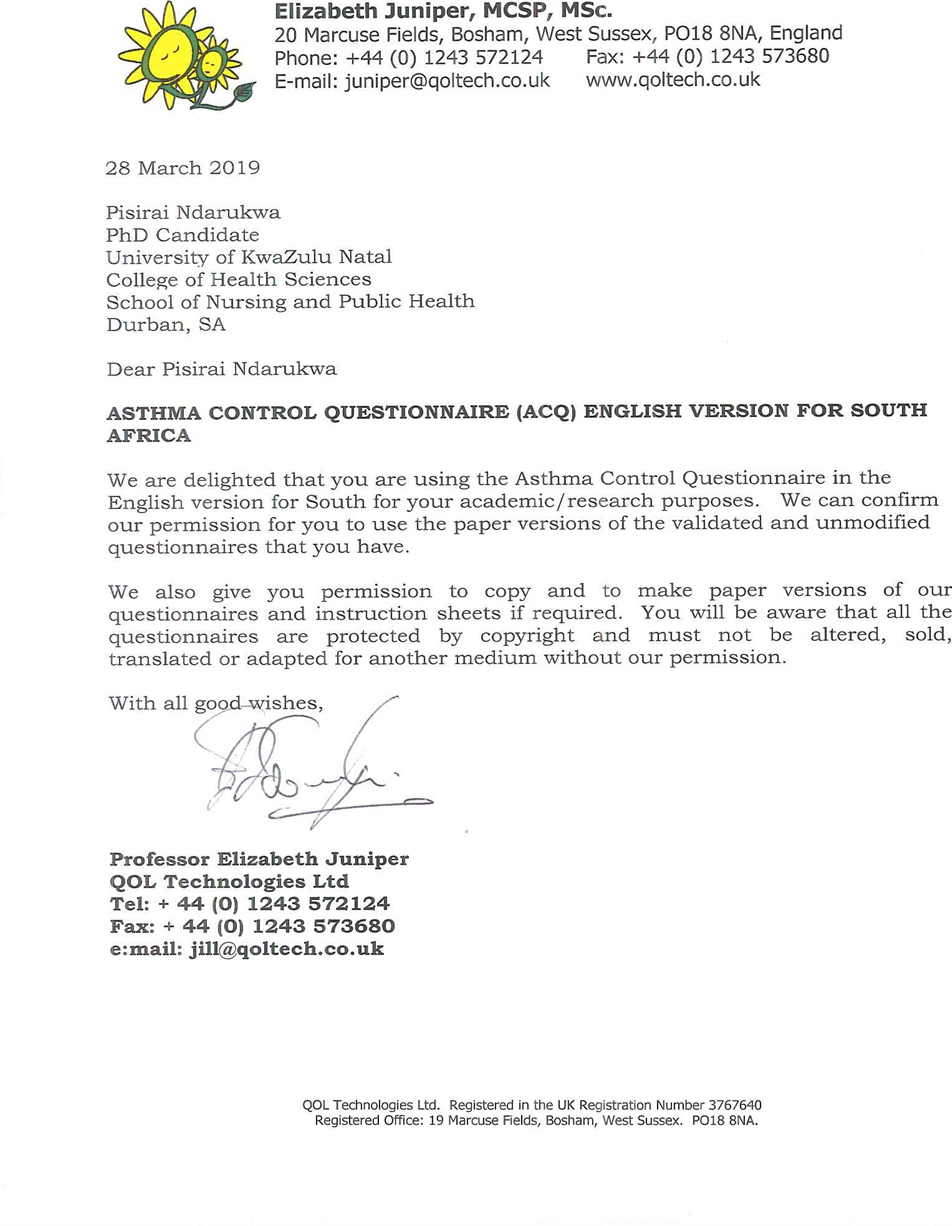
Appendix S2: ACQ letter of copyright permission.
